# Combining mutations at genes encoding key enzymes involved in starch synthesis affects the amylose content, carbohydrate allocation and hardness in the wheat grain

**DOI:** 10.1111/pbi.12908

**Published:** 2018-04-17

**Authors:** Ermelinda Botticella, Francesco Sestili, Francesca Sparla, Stefano Moscatello, Lucia Marri, Jose A. Cuesta‐Seijo, Giuseppe Falini, Alberto Battistelli, Paolo Trost, Domenico Lafiandra

**Affiliations:** ^1^ Department of Agriculture and Forestry Science University of Tuscia Viterbo Italy; ^2^ Department of Pharmacy and Biotechnology FABIT University of Bologna Bologna Italy; ^3^ National Research Council CNR‐Istituto di Biologia Agroambientale e Forestale Porano Terni Italy; ^4^ Carlsberg Research Laboratory Copenhagen Denmark; ^5^ Department of Chemistry ‘G. Ciamician’ University of Bologna Bologna Italy

**Keywords:** amylose, carbohydrates allocation, cell wall polysaccharides, gene expression, kernel hardness, resistant starch

## Abstract

Modifications to the composition of starch, the major component of wheat flour, can have a profound effect on the nutritional and technological characteristics of the flour's end products. The starch synthesized in the grain of conventional wheats (*Triticum aestivum*) is a 3:1 mixture of the two polysaccharides amylopectin and amylose. Altering the activity of certain key starch synthesis enzymes (GBSSI, SSIIa and SBEIIa) has succeeded in generating starches containing a different polysaccharide ratio. Here, mutagenesis, followed by a conventional marker‐assisted breeding exercise, has been used to generate three mutant lines that produce starch with an amylose contents of 0%, 46% and 79%. The direct and pleiotropic effects of the multiple mutation lines were identified at both the biochemical and molecular levels. Both the structure and composition of the starch were materially altered, changes which affected the functionality of the starch. An analysis of sugar and nonstarch polysaccharide content in the endosperm suggested an impact of the mutations on the carbon allocation process, suggesting the existence of cross‐talk between the starch and carbohydrate synthesis pathways.

## Introduction

Plants that accumulate large amounts of starch in their seeds, tubers, or roots constitute a primary source of calories for the human diet and also represent a source of raw feedstock for a diverse set of industrial products. Starch is a mixture of the two polysaccharides amylose—an essentially linear polymer—and the highly branched amylopectin (Shevkani *et al*., 2017). Their distinctive properties are responsible for the variation in functionality of starches obtained from different sources. In conventional bread wheats (*Triticum aestivum*), in which starch represents 70%–80% of the grain's endosperm, the two polysaccharides are present in a ratio of around 1:3. Amylose‐free wheat starch (colloquially referred to as ‘waxy’) is more crystalline than wild‐type starch, is more readily degraded by amylases, and absorbs and retains more water. These characteristics increase the utility of waxy wheats both in the formulation of animal feed and in the production of biofuel. In chilled and frozen dough‐based food products, the inclusion of waxy wheat flour offsets some of the decline in quality experienced during storage (Graybosch, 1998), while its positive effect on water holding ability has been promoted as a means of slowing the staling of bread (Jongsutjarittam and Charoenrein, 2014; Morita *et al*., 2002). While starch represents the predominant source of dietary carbohydrates, an excessive intake of calories has a negative impact on health. Thus, in food products, a high content of amylose is considered to be desirable, given that it makes the product less easily digestible. In addition, the structure of amylose is more resistant than that of amylopectin to the action of pancreatic enzymes, leaving it intact as a substrate for the gut microflora in the large intestine, which in turn boosts the production of short chain fatty acids. So‐called ‘resistant’ starch is currently being promoted in the context of addressing a range of human health issues (Lockyer and Nugent, 2017).

Several wheat improvement programmes have sought to alter end‐use quality by targeting genes in the starch synthesis pathway, either by exploiting naturally occurring allelic variants or by attempting to induce novel ones via mutagenesis or transgenesis. Wheats accumulating a less than normal proportion of amylose in their endosperm starch have been generated by knocking out the genes encoding granule‐bound starch synthase I (GBSSI), an enzyme responsible for the elongation of amylose chains. Because bread wheat is hexaploid, independent mutations at each of the three homeoloci *GBSSI‐A1*,* GBSSI‐B1* and *GBSSI‐D1* have to be combined into a single line by conventional breeding (Nakamura *et al*., 1995; Slade *et al*., 2005). Depending on their content of *GBSSI* mutations, the proportion of amylose in the endosperm starch has been successfully reduced from 25% to 30% all the way to 0% (Inokuma *et al*., 2016). High amylose content alleles are also available in a number of cereals, including wheat. The relative content of amylose can be boosted most easily by compromising the synthesis of amylopectin. Three enzymes are involved in amylopectin synthesis, namely: starch synthase (SS), which acts to elongate linear chains; starch branching enzyme (SBE), which promotes chain branching; and starch debranching enzyme (DBE), which trims the chains to ensure a suitable starch granule crystalline structure. The inactivation of SSIIa has been shown to increase the amylose proportion to between 37% (Yamamori *et al*., 2000) and 44% (Konik‐Rose *et al*., 2007; Luo *et al*., 2015). The suppression of SBEII activity is particularly effective in boosting the amylose content (Regina *et al*., 2006; Sestili *et al*., 2010a; Slade *et al*., 2012). Knocking out both *SBEI* and *SBEII* resulted in barley and wheat lines in which the endosperm starch was composed almost exclusively of amylose (Carciofi *et al*., 2012; Regina *et al*., 2015).

Here, a description is given of the identification and characterization of three wheat lines in which the grain amylose content varies from 0% to 79%. The lines were bred by combining mutations at all three homoeologs encoding either GBSSI, SSIIa, or SBEIIa, based on independent induced mutants for each locus, as described elsewhere (Botticella *et al*., 2011; Sestili *et al*., 2010b). The impact of varying the amylose level on the starch's physicochemical structure and functionality is explored, along with its effect on the composition of nonstarch polysaccharides and sugars.

## Results

### Production of the three amylose mutants Cad‐GBSSI*, Cad‐SSIIa* and Cad‐SBEIIa*

Here, we produced a set of three mutants targeting three key genes in starch synthesis, respectively *GBSSI*,* SSII*a and *SBEIIa* in the wheat varietal background cv Cadenza. For each gene, the lines carrying the null mutations at each homoeologous gene were combined generating the three *null* genotypes GBSSI *null* (Cad‐GBSSI*), SSIIa *null* (Cad‐SSIIa*) and SBEIIa *null* (Cad‐SBEIIa*) (Figure S1). As expected, the transcript abundance of the mutated gene was strongly reduced in Cad‐GBSSI* and Cad‐SBEIIa*, while the abundance of *SSIIa* was comparable to that in cv. Cadenza (see paragraph ‘The transcription of genes associated with carbon allocation in the endosperm’). Nonetheless, the analysis of starch granule proteins confirmed the absence of the SSIIa isoforms in Cad‐SSIIa* (data not shown).

As expected, the mutant lines generated in this work contain widely different levels of amylose in their grains. In respect to the amylose content of the cv. Cadenza (34% of total starch in grains), GBSSI mutant had no detectable amylose, while SSIIa and SBEIIa mutants were amylose rich (46% and 79% of total starch, respectively).

### Grain phenotypic traits and physicochemical properties of starch

**Figure 1:**
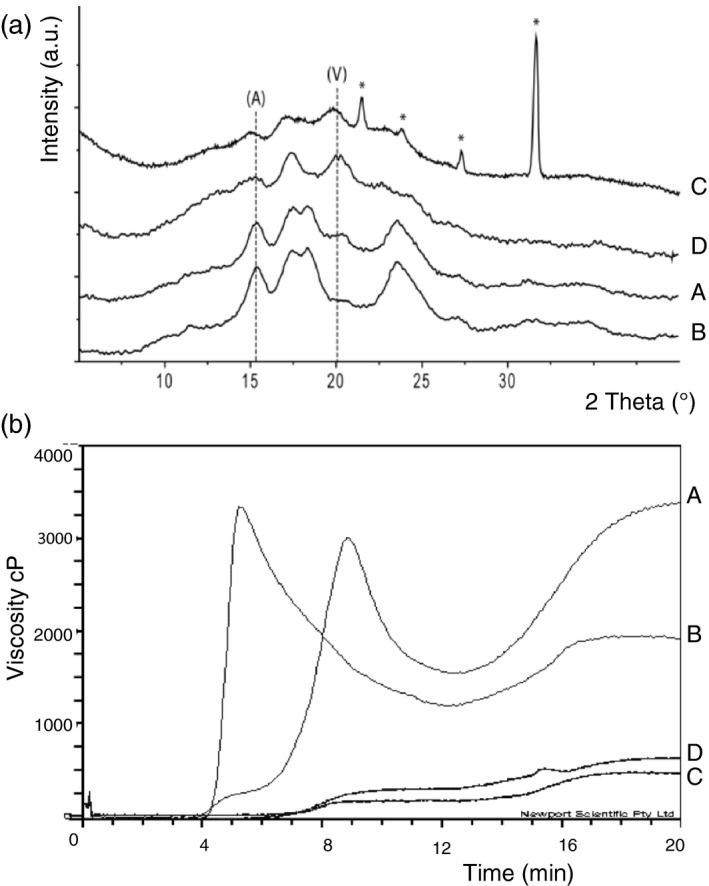
Crystallinity and viscosity in the mutant wheats and the control Cadenza. (a) X‐ray diffraction patterns of starch synthesized by (A) cv Cadenza (B) Cad‐GBSSI* (C) Cad‐SSIIa* and (D) Cad‐SBEIIa*. Peaks diagnostic for A‐ and V‐type crystals are indicated. The intensity is given in arbitrary units (a. u.). The asterisks indicate the presence of impurities in the sample. (b) Viscosity profiles as measured by RVA (A) cv Cadenza, (B) Cad‐GBSSI*, (C) Cad‐SSIIa* and (D) Cad‐SBEIIa*.

**Figure 2:**
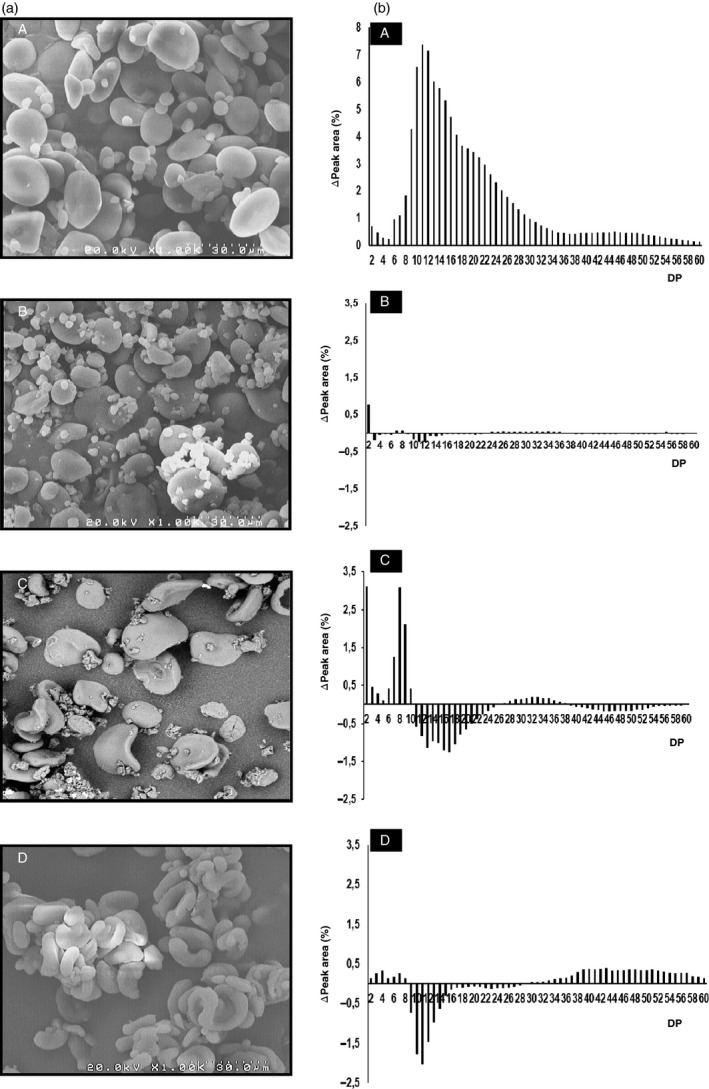
Starch granule morphology and amylopectin branched chains length in the set of mutant genotypes and the control. (a) Scanning electron micrographs of starch granules in flour prepared from (A) cv. Cadenza, (B) Cad‐GBSSI*, (C) Cad‐SSIIa* and (D) Cad‐SBEIIa*. (b) (A) Chain length distribution of amylopectin in the control cv Cadenza; the histograms illustrate the differences in chain length distribution between cv. Cadenza and the mutant lines and: (B) Cad‐GBSSI*, (C) Cad‐SSIIa* and (D) Cad‐SBEIIa*.

Grains of the cv. Cadenza have an average weight of 47 mg and 59% of this weight is constituted by starch. One‐third of total starch is amylose (34%; Table 1). The content of resistant starch, determined as percentage of whole flour, was negligible. Crystallinity of starch was determined by X‐ray powder diffraction analysis and 46% of starch was found in crystal form with a predominant A‐type packing (78%) and almost undetectable V‐type packing (Figure 1). Starch granules, as visualized by scanning electron microscopy, showed the typical bimodal distribution into large ellipsoidal granules (A‐granules) and small spherical ones (B‐granules) (Figure 2).

**Table 1:** Variation in grain and starch parameters among the three mutant lines and cv Cadenza. DSC: differential scanning calorimetry; HKW: hundred kernel weight; Values associated with a different letter differ from one another (*P* < 0.05)

Seed traits						DSC	X‐ray	
Genotype	HKW (g)	Hardness index	Total starch (%)	Resistant Starch (%)	Amylose (%)	*T*_0_ (°C)	Total crystallinity (%)	A‐type crystallinity (%)
Cadenza	4.7 ± 0.4^a^	61.8 ± 18.2^a^	58.9 ± 0.1^a^	0.2 ± 0.3^a^	34.0 ± 0.9^a^	57.3 ± 0.4^a^	46	78
Cad‐GBSSI*	4.5 ± 0.2^a^	73.5 ± 13.2^b^	57.2 ± 0.6^a^	0.2 ± 0.1^a^	nd	58.6 ± 0.9^a^	54	93
Cad‐SSIIa*	3.3 ± 0.3^b^	80.5 ± 15.0^c^	38.6 ± 0.7^b^	1.4 ± 0.1^b^	45.7 ± 1.0^b^	49.8 ± 0.3^b^	43	30
Cad‐SBEIIa*	4.2 ± 0.5^a^	86.1 ± 17.8^d^	57.0 ± 0.9^a^	7.2 ± 0.2^c^	78.7 ± 1.1^c^	58.4 ± 0.4^a^	39	32

Despite the absence of amylose, the grains of Cad‐GBSSI* displayed several phenotypic traits that were similar to those of the cultivar Cadenza, including the average grain weight, the total starch content and the content of resistant starch (Table 1). Amylopectin structure, as obtained by HPAEC‐PAD analysis, was also not qualitatively different from that found in Cadenza grains in terms of length and relative abundance of the chains (Figure 2). Starch granule morphology (SEM) was apparently normal (Figure 2). On the other hand, starch crystallinity of Cad‐GBSSI* was higher (54% vs. 46% of wild‐type starch) and the A‐type of crystal packing was largely predominant (93% vs. 78%) (Table 1). According to an SKCS analysis, hardness of the grain was also higher than in wild‐type seeds (+18%; Table 1). Moreover, the differential scanning calorimetry (DSC) analysis showed a slightly higher gelatinization temperature (*T*_0_ + 2%) for the starch isolated from Cad‐GBSSI* compared with the one isolated from the cv. Cadenza (Table 1), as expected for a more crystalline starch (Yasui *et al*., 1996). The flour made from Cad‐GBSSI* had also a different viscosity, showing a higher peak viscosity (PV) but lower final viscosity (FV) compared with the wild type (Figure 1 and Table S3).

Grains of Cad‐SSIIa*, containing starch that is moderately enriched in amylose (46% of total starch), were smaller in weight (−30%) and contained less total starch (−33%) than Cadenza grains (Table 1). The increased amount of amylose correlated with a 7‐fold increase in resistant starch (Table 1). The amylopectin produced by Cad‐SSIIa* was enriched in short chains (DP = 2–9) but deprived of longer chains with DP lying in the 11–22 range, compatible with a role of SSIIa in elongating the short chains (2–9 DP) into medium ones (11–22 DP; Figure 2). Starch granules morphology was also deeply altered: A‐granules were heavily distorted and were no longer recognizable as ellipsoidal in shape, while small B‐type granules were less abundant and not as spherical as in the typical Cadenza starch (Figure 2). Although the percentage of starch crystallinity was unaffected in Cad‐SSIIa*, the type of packing was predominantly V‐type, the A‐type of packing being less represented (30%; Figure 1; Table 1). Grain hardness was also increased (+30%; Table 1). The gelatinization temperature of the starch isolated from Cad‐SSIIa* was lower than that of Cadenza starch (Table 1), as also observed in an equivalent SSIIa mutant in barley (Morell *et al*., 2003). The rapid visco analysis (RVA) profile of the amylose‐rich, SSIIa mutant flour was flattened in respect to the Cadenza RVA profile, possibly reflecting the ability of amylose to restrict both water absorption and starch granule swelling (Figure 1).

For Cad‐SSIIa*, a further limit to the increase in viscosity could be represented by the decreased percentage of total starch content.

Grains of Cad‐SBEIIa* were the most enriched in amylose (79% of total starch, Table 1). The hardness index was also very high (+38%) but the total starch content and the average seed weight were unaffected by the mutation. The amylose enrichment led, as expected, to a strong increase in resistant starch (36‐fold vs. Cadenza) (Table 1). The residual amylopectin found in Cad‐SBEIIa* varied significantly from Cadenza starch showing a marked decrease in intermediate chains (DP = 9–16), and a corresponding increase in very long chains (DP > 30) indicating that in the absence of SBEIIa activity, intermediate chains are abnormally elongated to very long ones (Figure 2). Starch crystallinity dropped to 39% and crystal packing was predominantly V‐type and only partially (32%) A‐type, like in Cad‐SSIIa* but opposite to control starch (Table 1). Similar to Cad‐SSIIa*, large (A‐type) starch granules were deformed in Cad‐SBEIIa* as well, and small B‐type granules were almost absent (Figure 2). The starch obtained from Cad‐SBEIIa* mutant grains displayed higher gelatinization temperature (Table 1), as also observed for the equivalent mutant in barley (Regina *et al*., 2010). Moreover, the conventional transition, attributable to the dissociation of amylopectin chains, was replaced by a flatter peak possibly due to the transition of the amylose‐lipid complex (Regina *et al*., 2010). Similar to Cad‐SSIIa*, also Cad‐SBEIIa* produced a flour with altered viscosity because amylose can limit water absorption and starch granule swelling and thereby lead to a flattened RVA profile (Figure 1).

### Nonstarch carbohydrates

The most abundant nonstarch carbohydrates present in wild‐type grains were fructans (on average representing 2.8% of the grains’ dry matter), water‐soluble alpha‐glucans (2.05%) and sucrose (1.7%). The remaining soluble carbohydrates constituted together <1% of the grains’ dry matter (Table 2).

**Table 2:** Variation in the content of carbohydrates and sugars among the three mutant lines and cv Cadenza. TOT‐AX: total arabinoxylans; WE‐AX: water‐extractable arabinoxylans; WS α‐glucan: water‐soluble α‐glucan. Values associated with a different letter differ from one another (*P* < 0.05)

Genotype	Fructans (mg/g)	TOT‐AX (%)	WE‐AX (%)	β‐Glucans (%)	WS α‐glucan (mg/g)	Glucose (mg/g)	Fructose (mg/g)	Sucrose (mg/g)	Galactose (mg/g)	Raffinose (mg/g)	1‐ketose (mg/g)	Maltose (mg/g)
Cadenza	20.2 ± 2.8^b^	4.0 ± 0.3^a^	0.6 ± 0.0^a^	0.6 ± 0.0^a^	20.5 ± 2.9^b^	0.35 ± 0.05^b^	0.53 ± 0.03^b,c^	11.4 ± 0.1^c^	0.02 ± 0.00^b^	4.3 ± 0.2	1.7 ± 0.1^c^	0.37 ± 0.02^c^
Cad‐GBSSI*	23.7 ± 1.4^b^	4.8 ± 0.2^a^	0.7 ± 0.0^a^	1.0 ± 0.1^b^	32.9 ± 1.8^a^	0.45 ± 0.02^b^	0.50 ± 0.02^c^	14.7 ± 0.2^b^	0.04 ± 0.00^a^	4.6 ± 0.1	2.4 ± 0.2^b^	0.54 ± 0.02^b^
Cad‐SSIIa*	42.2 ± 0.7^a^	6.8 ± 0.1^b^	0.9 ± 0.0^b^	1.6 ± 0.4^d^	15.3 ± 0.7^b,c^	1.03 ± 0.03^a^	0.66 ± 0.04^a^	25.9 ± 0.5^a^	0.04 ± 0.00^a^	6.7 ± 1.2	4.2 ± 0.3^a^	1.75 ± 0.07^a^
Cad‐SBEIIa*	25.4 ± 3.7^b^	5.2 ± 0.3^a^	1.3 ± 0.0^d^	1.0 ± 0.1^b^	13.6 ± 0.6^c^	0.32 ± 0.05^b^	0.62 ± 0.03^a,b^	14.4 ± 0.3^b^	0.03 ± 0.00^b^	5.5 ± 0.2	2.5 ± 0.1^b^	0.19 ± 0.05^d^

**Figure 3:**
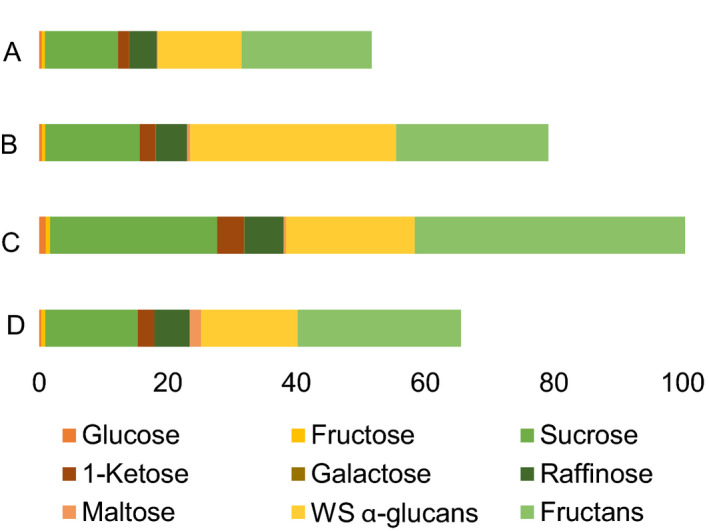
Total soluble carbohydrate content in the flour of cv. Cadenza and its derived null mutant lines: (A) cv. Cadenza, (B) Cad‐GBSSI*, (C) Cad‐SSIIa* and (D) Cad‐SBEIIa*. The full bar represents the total soluble carbohydrate content expressed as mg/g. The composition of the soluble carbohydrates is reported as the sum of glucose, fructose, 1‐kestose, sucrose, raffinose, maltose, galactose, water‐soluble α‐glucans and fructans.

Nonstarch carbohydrates including soluble sugars were generally higher in mutant lines than in wild‐type seeds. Total soluble carbohydrates were twice as much in Cad‐SSIIa*, in which starch was lower than in all other genotypes, but total soluble carbohydrates were higher also in Cad‐GBSSI* and Cad‐SBEIIa* mutants with normal levels of starch (Figure 3). In more detail, amylose‐free Cad‐GBSSI* mutant was enriched between 1.3‐ and 2‐fold in β‐glucans, water‐soluble alpha‐glucans, sucrose, galactose, 1‐kestose and maltose, but contained wild‐type levels of fructans, arabinoxylans (either total or water‐extractable) and sugars like glucose, fructose and raffinose (Table 2). In Cad‐SSIIa*, all nonstarch carbohydrates were increased (1.7‐ to 2.7‐fold): fructans, arabinoxylans (total and water‐extractable) and β‐glucans. A similar but more pronounced trend was observed for soluble sugars, with maltose (4.7‐fold) and glucose (3.4‐fold) increasing the most, but 1‐ketose, sucrose and galactose also increasing 2‐fold or more (Table 2). Cad‐SBEIIa* showed the highest value of water‐extractable arabinoxylans (1.3%), and also β‐glucans and sucrose were increased significantly, but all other metabolites remained at wild‐type levels or below (maltose and water‐soluble alpha‐glucans; Table 2).

### The transcription of genes associated with carbon allocation in the endosperm

The expression of a set of genes involved in starch and nonstarch carbohydrates metabolism in wheat grains was evaluated by qRT‐PCR in wild type, Cad‐GBSSI*, Cad‐SSIIa* and Cad‐SBEIIa*. Starch biosynthesis initially involves sucrose synthase (Susy), UDP‐glucose pyrophosphorylase (UGPase) and ADP‐glucose pyrophosphorylase (AGPase) for the conversion of sucrose into ADP‐glucose. AGPase exists in two isoforms in wheat endosperm, the cytosolic isoform is coded by genes *AGPaseL1* and *AGPaseS1*, the plastidic isoform by genes *AGPaseL2* and *AGPaseS2*. Both isoforms are made of large (L) and small (S) subunits. An ADP‐glucose transporter (ADPG‐t) is required for the import of cytosolic ADP‐glucose into the amyloplast for starch biosynthesis.

**Figure 4:**
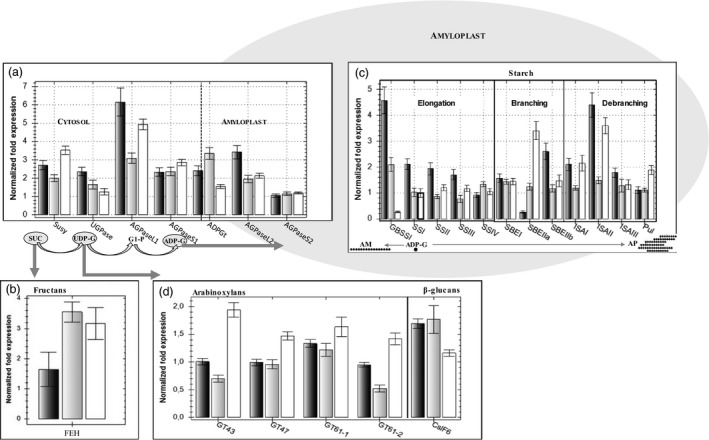
The transcription of genes encoding key enzymes in carbon allocation in the endosperm as estimated by qRT‐PCR. Cad‐SBEIIa* is shown by black bars, Cad‐SSIIa* by grey bars and Cad‐GBSSI* by white bars. Genes involved in (a) sugar cleavage and cytosolic synthesis of ADP‐glucose, (b) 6&1 fructan exohydrolase (6&1FEH), (c) starch synthesis in the amyloplast, (d) arabinoxylan and β‐glucan synthesis. Each bar represents the mean of three biological replicates and standard errors are indicated by thin black bars. The level of expression of the control is 1.

The expression level of the genes involved in the biosynthesis of the starch precursor ADP‐glucose was in most cases higher in the three null mutant lines than in the wild type, and no gene was found repressed (Figure 4). *Susy* and three *AGPase* genes (*AGPaseL1*,* S1*,* L2*) were up‐regulated more than 2‐fold in Cad‐GBSSI*. Two *AGPase* genes (*AGPaseS*1 and *L2*) were significantly up‐regulated also in Cad‐SSIIa*, together with *ADPG‐t* coding for the ADP‐glucose transporter. In Cad‐SBEIIa*, all genes were up‐regulated more then 2‐fold except for the gene coding the small subunit of plastidic AGPase whose expression was genotype‐independent (Figure 4).

Inside the amyloplast, ADP‐glucose is polymerized by GBSSI to amylose. Amylopectin formation and modelling require a large set of enzymes belonging to starch synthase (SSI, SSII, SSIII, SSIV), starch branching enzyme (SBEI, SBEIIa, SBEIIb) and starch debranching enzyme families (ISAI, ISAII, ISAIII, Pul). The expression of some of these genes was not significantly affected by the genotype (*SSIII*,* SSIV*,* SBEI*,* ISAIII*,* Pul*).

On the other hand, few genes were significantly (>2‐fold) up‐regulated in mutants. The transcripts for one starch branching enzyme (SBEIIa, 2.7‐fold) and two debranching enzymes (ISAI, ISAII) were found increased in the amylose‐free Cad‐GBSSI* mutant. On the contrary, *GBSSI* was up‐regulated in the amylose‐rich Cad‐SSIIa* mutant. *GBSSI* up‐regulation was even more pronounced (4.5‐fold) in Cad‐SBEIIa* containing the highest levels of amylose, and genes involved in the metabolism of amylopectin such as *SSI*,* SBEIIb* (possibly compensating the lack of *SBEIIa*), *ISAI* and *ISAII* were all strongly up‐regulated (Figure 4).

Besides starch, sucrose is also the precursor of fructan biosynthesis. The transcription intensity of the two fructan synthesis genes (1‐SST and FFT 6SST) was too low in the developing grain to reveal any genotypic variation. Gene FEH was selected as a representative of the genes encoding fructan exohydrolases. This gene is known to be transcribed at appreciable levels in the maturing grain (Cimini *et al*., 2015) and was found to be up‐regulated in both Cad‐GBSSI* and Cad‐SSIIa*.

The biosynthesis of arabinoxylans and β‐glucans depends on the availability of UDP‐glucose and involves genes encoding various glycosyl transferases (GT‐43, GT‐47, GT‐61‐1 and GT‐61‐2) whose expression was determined in the grains of the three null mutant lines and in the cv. Cadenza. None of these genes were significantly (<2‐fold) up‐ or down‐regulated in any genotype, although expression of all of them tend to be consistently higher in Cad‐GBSSI* and possibly lower in Cad‐SSIIa* (Figure 4).

With respect to the gene encoding CSLF6, the major enzyme involved in β‐glucan synthesis, which also relies on UDP‐glucose availability, its transcript abundance was apparently higher in amylose‐rich mutants, but the increase was less than 2‐fold (Figure 4).

## Discussion

### The effect of the mutations on the structure and composition of starch

**Figure 5:**
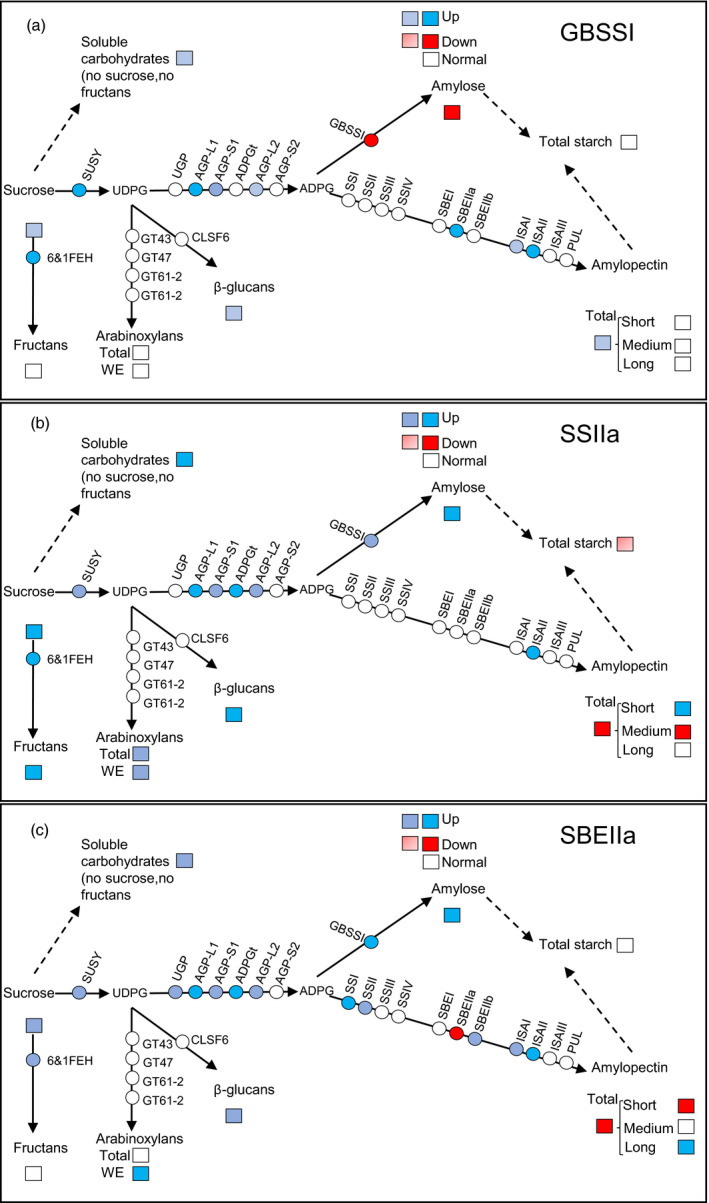
A schematic illustration of metabolic flux and of the transcripts level of the genes involved in the pathways of sucrose, starch, β‐glucans, arabinoxylans and fructans in the endosperm of the three mutant lines: panel (a) Cad‐GBSSI*; panel (b) Cad‐SSIIa*; and panel (c) Cad‐SBEIIa*. Squares indicate metabolites; circles stand for genes. Dashed arrows indicate the overall flux whereas the solid arrows stand for the single reaction. Full colours indicate, for genes, increase in expression of 3‐fold or more (blue), and decrease of expression of 3‐fold or less (red); for metabolites, statistically significant changes of +100% or more (blue), or −50% or less (red). Thresholds for intermediate colours were: increase in gene expression (between 2‐ and 3‐fold; light red), decrease in gene expression (between 2‐ and 3‐fold); metabolites: light blue when the increase was significant but less than 100%; light red when significant but less than −50%.

The suppression of *GBSSI*,* SSIIa* and *SBEIIa* genes deeply influences the structure and composition of the starch, the carbon allocation and the expression of key genes involved in sugar and carbohydrate metabolism (Figure 5).

The silencing of *SBEIIa* homeologs is known to enhance the proportion of amylose in the starch deposited in the wheat endosperm (Regina *et al*., 2015; Slade *et al*., 2012). The RNAi‐induced knock‐down of *SBEIIa* results in a starch composed of 88.5% amylose (compared to 31.8% in wild‐type starch; Regina *et al*., 2015), while according to Slade *et al*. (2012), segregants which lacked functional copies of all three homeologs produced starch with an amylose content of 55%, compared to just 23% in the starch produced by wild‐type segregants. Similarly, knocking down *SBEIIa* in durum wheat resulted in a tripling of the proportion of the starch represented by amylose (Sestili *et al*., 2010a,b); while the targeting of the same genes by a TILLING approach was responsible for a minor increase in the amylose percentage (+94%–96%) (Sestili *et al*., 2015; Slade *et al*., 2012). Here, the amylose content of the endosperm starch formed by Cad‐SBEIIa* was 79%, compared with 34% in cv. Cadenza, similarly representing an approximate doubling. The variation in amylose content observed among these various *SBEIIa* deficient lines could reflect an effect of genetic background, differing methods of estimating amylose content, residual SBEIIa activity and/or off‐target gene silencing. In particular, the use of RNAi to silence *SBEIIa* also impedes the expression of *SBEIIb* genes, thereby explaining, at least in part, the higher amylose contents achieved in these lines compared to what has been achieved by mutagenesis. Consistently, here we show that the knock out of the three *SBEIIa* homeologous genes is partially compensated by of the up‐regulation of SBEIIb in Cad‐SBEIIa* (Figure 4). The collateral silencing of different SBE isoforms has a similar incremental effect on amylose contents in other cereal species as well (Carciofi *et al*., 2012; Regina *et al*., 2015).

As anticipated, raising the amylose content of the starch made it more resistant to degradation. The proportion of the endosperm starch which was resistant to degradation in Cad‐SSIIa* and Cad‐SBEIIa* reached 1.4% and 7.2% of whole flour, respectively; levels which should be of interest to food manufacturers concerned with reducing the glycemic index of their products, as well as to dietitians treating various eating disorders (Lafiandra *et al*., 2014). The two mutant lines in which the amylose content was enhanced (Cad‐SSIIa* and Cad‐SBEIIa*) produced starch exhibiting an altered crystallinity type and distribution of amylopectin chain length. As has also been remarked by Yasui *et al*. (1996, 2009), the loss‐of‐function of *GBSSI* had negligible effect on the distribution of amylopectin chain length. Note that no conclusion can be drawn regarding the effect (if any) on the representation of extra‐long chains (DP > 77) in Cad‐GBSSI*, as these were not assayable using the present detection system: other SSs may act to form such extra‐long chains when GBSSI is absent (Yoo and Jane, 2002).

The crystalline form of the starch formed by Cad‐GBSSI* was predominantly A‐type (as expected in starch dominated by amylopectin), while the other two null mutant lines’ starch was deficient in A‐type crystallinity (and enriched in V‐type). The pattern of crystallinity seen in the starch of Cad‐SBEIIa* and Cad‐SSIIa* has also been noted in high amylose rice and barley starch (Asai *et al*., 2014; Nishi *et al*., 2001; Sparla *et al*., 2014). The absence of SSIIa activity leads to an enrichment of short chains (DP of 2–9) and a loss in the representation of medium length ones (DP of 12–25) (Luo *et al*., 2015; Yamamori *et al*., 2000). This change has an effect on the structure of amylopectin, in that the packing of chains is compromised, resulting in the distortions to granule morphology observed by scanning electron microscopy (Figure 2). In starch made by Cad‐SBEIIa*, the absence of short amylopectin double helices (correlated with A‐type crystallinity) is likely due to a deficiency in DP 6–17 chains, while the increased representation of long chains (DP > 24) produces a structure which resembles that of amylose linear chains. A similar amylopectin structure has been described by Regina *et al*. (2015) in wheats deficient for *SBEIIa* transcript.

### The effect of the mutations on grain phenotype

Differences between the two enhanced amylose lines were revealed with respect to both grain weight and total starch content. The suppression of *SSIIa* was associated with a substantial decrease in both traits, thereby compromising economic yield, as has been documented not just in wheat, but also in some other cereals (Botticella *et al*., 2016; Kosar‐Hashemi *et al*., 2007; Luo *et al*., 2015; Morell *et al*., 2003; Sparla *et al*., 2014). As a matter of fact, despite the high amylose of Cad‐SSIIa*, the amylose content of mutant grains on a fresh weight basis is lower than that of the wild type because of the decrease in total starch. The general implication is that in the absence of SSIIa activity, amylopectin synthesis cannot be rescued by the activity of other SS enzymes, and that amylose synthesis can only partially compensate for the inhibited amylopectin synthesis.

In both Cad‐GBSSI* and Cad‐SBEIIa*, in contrast*,* neither grain weight nor total starch content was affected. Thus, in Cad‐GBSSI*'s starch, the loss in amylose was effectively replaced by a compensatory increase in the amylopectin fraction. Increasing the amylose content of the endosperm starch is known to alter the texture of the grain (Hogg *et al*., 2017; Schönhofen *et al*., 2017). According to Schönhofen *et al*. (2017), a wheat line defective for both SBEIIa and SBEIIb forms grains which are 25% harder than normal, and mutations to *SSIIa* also convert a wheat line from a soft‐ to a hard‐grained type (Hogg *et al*., 2017). Similarly, Clarke *et al*. (2008) have noted the increase in grain hardness realized by knocking out *SSIIa* in barley. Here, all three null mutant lines formed harder grains than cv. Cadenza. Grain texture in bread wheat is strongly determined by an interaction between purindolines and polar lipids adsorbed on the surface of the starch granules. In most hard‐grained varieties, the level of purindoline is low, resulting in a minimal interaction (Bhave and Morris, 2008). This is unlikely to form the basis of the change in grain texture in present mutants. Instead, the basis could involve one or more of protein content, vitreousness, grain size, the content of water‐soluble pentosans, moisture and free polar lipids (Bhave and Morris, 2008). Given that Cad‐GBSSI* also produced hard grains, a high amylose content in itself cannot underlie grain hardness, but the absence of free polar lipids bound to the starch granules’ surface consequent to the lack of amylose may be important. Also, likely relevant is the increased deposition of water‐soluble arabinoxylans, here observed in both Cad‐SSIIa* and Cad‐SBEIIa*, but not in Cad‐GBSSI*. According to Gaines *et al*. (2000), starch formed from larger granules tends to form soft wheats, as a result of the formation of a less compact endosperm. It is likely also that granule morphology exerts an influence over grain structure, as the deformed granules seen in the SSIIa* and SBEIIa* mutant line may act to enhance their embedding within the protein matrix, thereby reducing the voids in the seed and favouring the development of a harder endosperm; this hypothesis is consistent with the major difficulty experienced in isolating starch granules from Cad‐SSIIa*.

### Pleiotropic effects of the mutations on the content of nonstarch carbohydrates in the endosperm

Altering through mutagenesis, the nature of the starch synthesized in the wheat endosperm was also associated with changes to its sugar and nonstarch carbohydrate composition. In Cad‐SSIIa*, the soluble sugar content was significantly increased, while that of starch was decreased (Figure 5). The general understanding is that cereal grains developing under nonstressed growing conditions are not source limited (Jenner *et al*., 1991; Weichert *et al*., 2010), so the decrease in sink capacity in term of starch accumulation is partially compensated by an increase in nonstarch carbohydrates (particularly fructans, arabinoxylans and β‐glucans) derived from sucrose translocated into the endosperm. The compensation is partial, however, because soluble carbohydrates may totally account for only a fraction (1/4) of the total starch missing. There were no differences in the content of soluble carbohydrate in the immature grains of any of three mutants (data not shown), so the indication is that the development of an inadequate sink must occur relatively late during the grain filling process. SSIIa appeared, from current observations, to be a key enzyme for determining both the composition of the starch and the balance of sugars, β‐glucans, arabinoxylans and fructans deposited in the endosperm. Li *et al*. (2017) have recently described a similar accumulation of fructans in a SSIIa mutant line of bread wheat, while the equivalent mutant in durum wheat induces a significant increase in the endosperms’ β‐glucan and arabinoxylan content (Botticella *et al*., 2016).

Targeting starch synthesis affects other aspects of carbon utilization in Cad‐SBEIIa*.

In Cad‐GBSSI*, the ADP‐glucose no longer usable as a precursor of amylose was redirected towards amylopectin synthesis and total starch content remained constant; despite this, there was a marked alteration in the composition of sugars and soluble carbohydrates, which implies the existence of a network of strongly interconnected reactions. Starch synthesis is known to be regulated by protein complexes involving both synthesis‐related and nonrelated enzymes (Hennen‐Bierwagen *et al*., 2009); such protein–protein interactions could serve as a signalling mechanism in the GBSSI* mutant plants, governing the re‐partitioning of carbon. The composition of sugars and soluble carbohydrates changed also in Cad‐SBEIIa*, where opposite to Cad‐GBSSI*, ADP‐glucose was redirected from amylopectin to amylose synthesis, confirming the view that mutations that affect the amylose:amylopectin ratio may have secondary effects on the composition of soluble carbohydrates, even in the presence of normal starch levels.

### The effect of the mutations on sugar mobilization

The enzymes Susy, UGPase, AGPase and ADPG‐t are key control points of carbon partitioning, acting upstream of starch synthesis. Susy cleaves sucrose, thereby releasing UDP‐glucose, which is then converted to glucose‐1‐phosphate by UGPase. Alternatively, UDP‐glucose sustains the biosynthesis of arabinoxylans and β‐glucans. Glucose‐1‐phosphate acts as the substrate for cytosolic AGPase to synthesize ADP‐glucose, which is transported to the amyloplast by ADPG‐t. *Susy* was up‐regulated in all three of the present mutants, while the gene encoding UGPase was up‐regulated in Cad‐SBEIIa*, only marginally induced in Cad‐SSIIa* and not induced at all in Cad‐GBSSI* (Figure 4). According to Clarke *et al*. (2008), in a barley line lacking SSIIa activity, *Susy* is also up‐regulated. The genes encoding the small and large AGPase cytosolic subunits and ADPG‐t were up‐regulated in all three mutant lines, which suggests an increased rate of transport of ADP‐glucose to the amyloplast. It is known that the expression of AGPase is increased by sugars, thereby providing a way for starch accumulation to respond to changes in the plant's carbon status (Geigenberger, 2011). However, the observed increase in both *AGPase* transcript abundance did not lead to a detectable increase in the starch content of the mutants’ endosperms. In rice, it has been noted that over‐expressing a bacterial cytoplasmic‐localized AGPase has the effect of boosting the grains’ content of ADP‐glucose and certain upstream metabolites, but this additional carbon flux was not channelled into starch synthesis, possibly because the factor(s) limiting the amount of carbon which can be fixed as starch is located downstream of ADP‐glucose formation (Nagai *et al*., 2009). It has also been suggested that increases in the synthesis of ADP‐glucose (as well as of other hexose sugars) may be used as a signal to initiate the reprogramming of gene expression (Cakir *et al*., 2016). The over‐expression of genes encoding ADPG‐t has been shown to have an effect on the amylose/amylopectin ratio (Emes *et al*., 2003). As the affinity of GBSSI for ADP‐glucose is lower than that for soluble SSs, amylose synthesis tends to be favoured in the presence of high concentrations of ADP‐glucose, which could account for some of the observed increase in amylose:amylopectin ratios in the grain of Cad‐SSIIa* and Cad‐SBEIIa*; it agrees also with the increase in the level of transcription of *GBSSI*, which encodes the enzyme responsible for amylose synthesis (Figure 4).

### The effect of the mutations on the transcription of starch synthesis‐related genes

Cad‐GBSSI* featured a modest increase in the abundance of transcript generated from genes encoding all three classes of enzyme involved in amylopectin synthesis, namely the SSs, SBEs and DBEs. This up‐regulation, which was most pronounced for genes encoding branching enzymes and ISA, was consistent with the boost to amylopectin synthesis. Zhang *et al*. (2012) have reported similar transcriptional consequences in a GBSSI deficient rice mutant. In Cad‐SBEIIa*, both *GBSSI* and *ISAII* were up‐regulated. ISAII may contribute to amylose synthesis by providing α‐glucans to GBSSI for elongation. In Cad‐SSIIa*, there was no evidence for the up‐regulation of genes encoding other SSs. The rather modest increase in the transcript abundance of *SBE* and *DBE* genes observed in this mutant potentially reveals a feedback regulatory mechanism based on gene products downstream of SSIIa. The only responsive gene involved in the synthesis of the structural carbohydrates β‐glucan and the arabinoxylans was *ClSF6,* which was up‐regulated in Cad‐SSIIa*. This effect may represent a further consequence of the availability of carbon which would otherwise have been directed into the synthesis of amylopectin.

## Conclusions

Here, the consequences of abolishing either GBSSI, SSIIa, or SBEIIa on the composition of the wheat endosperm have been explored. The null mutant lines were established in a common, stabilized varietal background to allow for meaningful comparisons to be made. Flour variants with respect to the starch's amylose/amylopectin ratio are advantageous for certain food and nonfood products. The pleiotropic effects associated with the presence of each of these mutants involved some redistribution of carbon among the different fractions of carbohydrate accumulated in the endosperm. The grain yield penalty associated with SSIIa deficiency was compensated by the mutant endosperms ability to accumulate a higher amount of resistant starch, as well as of the health‐linked β‐glucans, arabinoxylans and fructans. Cad‐SBEIIa* was of particular interest, because it produced a sizable ratio of resistant starch, but did not suffer any major loss in grain weight.

## Experimental procedures

### Plant materials

Single null mutants for each of the *GBSSI, SSIIa* and *SBEIIa* homeologs were previously isolated in the cultivar (cv.) Cadenza, using the TILLinG (Targeting Induce Local lesions in Genome) procedure (Botticella *et al*., 2011; Sestili *et al*., 2010b). Intercrossing of the mutants was used to combine the mutations into a single line (Figure S1). F_2_ segregants lacking the products of either *GBSSI* or *SSIIa* were selected by SDS‐PAGE profiling of starch granule proteins (Sestili *et al*., 2010a,b); while the tetra ARMS PCR technique (Ye *et al*., 2001) was used to track the *SBEIIa* mutations (Table S1). Each of the triple null mutant lines was backcrossed to cv. Cadenza to reduce the content of nonrelevant mutations. The resulting selfed progenies were genotyped as above to derive homozygotes, and these selections were advanced by selfing to the F_5_ generation. The phenotype of the seeds for the three mutant lines has been shown in Figure S1.

### Starch extraction and starch granule morphology

Starch was isolated from whole flour using the dough ball method, in which a 5–10 g of wholemeal flour was mixed with 0.6 parts (w/w) of water and kneaded until it formed a dough. Starch was washed from the dough with 300 mL water, then, following a centrifugation (1500 g), the pellet was rinsed twice in 20 mL water. The thin yellow‐grey layer deposited on the surface of the starch pellet was removed with a spatula, and the starch left to dry for 2 days at 25 °C. For the SSIIa mutant, starch was isolated by crushing individual grains, followed by application of the starch granule extraction method (Zhao and Sharp, 1996); the resulting pellet was air‐dried and powdered to avoid its transition to a glassy state.

The morphology of starch granules was analysed using a scanning electron microscopy (SEM) Hitachi S‐4000 (Hitachi, Tokyo, Japan), as reported by Sparla *et al*. (2014).

### Single Kernel Characterization System

Single Kernel Grain hardness was determined by measuring crush force, using the Perten Single Kernel Characterization System (SKCS 4100), as reported by Botticella *et al*. (2016).

### Quantification of amylose, total starch, resistant starch and β‐glucan

The starchs’ amylose contents were determined from a 15 mg aliquot of purified starch using a colorimetric assay based on the iodine–amylose reaction (Chrastil, 1987) . A standard curve was generated from a mixture of potato amylose (Fluka, Neu‐Ulm, Germany) and wheat amylopectin (Sigma Aldrich, St. Louis, MO). Each value represented the mean of three technical replicates and each genotypic value the mean of three biological replicates. The contents of total starch, resistant starch and β‐glucan were determined from whole flour samples, using kits supplied by Megazyme (Irishtown, Ireland). The total starch contents were determined using the protocol specified for ‘samples containing also resistant starch’. For each of these variates, the readings represented the mean of three technical replicates, while each genotypic value was derived from the mean of three biological replicates.

### Quantification of arabinoxylan, fructan and sugars

Water‐extractable and total arabinoxylan contents were determined using a colorimetric method (Finnie *et al*., 2006). Fructan and nonstructural carbohydrates were extracted as reported by Cimini *et al*. (2015).

Soluble carbohydrates present in the supernatant were analysed using a Dionex ICS‐5000 high‐performance anion exchange chromatograph‐pulsed amperometric detector (HPAEC‐PAD) (Thermo Scientific, Sunnyvale, CA), consisting of an isocratic quaternary pump, a pulsed amperometric detector, an injection valve with a 15 mm^3^ injection loop and an analytical CarboPac Pa100 column (4 mm × 250 mm) with its guard column. The detection cell contained a gold working electrode (1.0 mm in diameter) and an Ag/AgCl reference electrode. Elution was carried out at 30 °C with a solute flow rate of 1 mL/min. Sugars were eluted as described by Cimini *et al*. (2015). The galactose and raffinose contents were measured following a slightly modified form of the protocol given by Santi *et al*. (2015): the analytical column used for this purpose was a CarboPac Pa210‐Fast–4, and the material was eluted in 12 mm NaOH at 30 °C with a flow rate of 0.8 mL/min. The sugars were identified on the basis of retention time, using appropriate standards (Sigma Aldrich, St. Louis, MO). The total fructan content was determined as described by Verspreet *et al*. (2012).

### Rapid visco analysis and differential scanning calorimetry

Wholemeal flour (three technical replicates) was subjected to RVA, as described by Sestili *et al*. (2010a). For the DSC, a 5–10 mg aliquot of starch, obtained as described above, was weighed in an aluminium pan and suspended in water (30% w/v). Standard wheat flour (Megazyme, Irishtown, Ireland) was used as a control. The pans were hermetically sealed and the samples allowed to equilibrate for 1 h at room temperature. Gelatinization temperatures were determined by heating the pan from 40 to 90 °C at 10 °C/min in a DSC‐1 STAR^e^ System device (Mettler Toledo, Columbus, OH).

### Chain branching pattern and crystallinity

A 2‐mg aliquot of starch, obtained as above, was used to determine the distribution of amylopectin chain length, based on HPAEC‐PAD analysis, modified from the method given by Sestili *et al*. (2016). After debranching, the supernatant was diluted to 0.4 mg/mL and a 10 μL aliquot was injected onto a CarboPac PA‐100 4 × 250 mm column, using a Dionex ICS 3000 system equipped with an autosampler (Thermo Fisher). The sample was eluted by imposing a linear gradient of 0–200 mm NaOAc in 50 mm NaOH over 10 min, followed by a convex gradient of 200–390 mm NaOAc over the subsequent 120 min, then by 1 m NaOAc for 5 min and finally by 0 mm NaOAc for 10 min. After baseline subtraction from water injections, peak areas were normalized to the total peak area of the injected sample. Starch crystallinity was determined from X‐ray powder diffraction patterns, as described by Sparla *et al*. (2014).

### RNA isolation and quantitative real‐time PCR (qRT‐PCR)

RNA was isolated using a Spectrum™ total plant RNA kit (Sigma Aldrich, St. Louis, MO) from snap‐frozen grains harvested 18 days postanthesis developing on field‐grown plants. Each genotype was represented by a bulk of four grains. One μg of the extracted RNA was used as template for the synthesis of cDNA, following the protocol of the QuantiTect Reverse Transcription Kit (Qiagen, Hilden, Germany). The subsequent qRT‐PCRs were run using a CFX 96 Real‐Time PCR Detection System device (Bio‐Rad, Hercules, CA), and the 10 μL reactions were formulated with SsoAdvUniver SYBR GRN SMX (Bio‐Rad). The reactions were initially denatured (94 °C/30 s), then cycled 40 times through the sequence 94 °C/5 s, 60 °C/30 s. A melting curve was generated over the range 65–95 °C, with 0.5 °C increments @ 5 s. Transcript quantification was enabled by CFX manager software. Each estimate of transcript abundance was based on the mean of three technical replicates, and each genotypic value was based the mean of two biological replicates. The list of genes analysed, along with the housekeeping gene, and the sequences used as primers are shown in Table S2.

### Statistical analysis

One‐way analyses of variance (ANOVAs) were conducted, using the *post hoc* Tukey HSD (Honestly Significant Difference) parameter to assess the significance of differences between means.

## Conflict of interest

The authors declare no conflict of interests.

## Supporting information


**Figure S1** Scheme of crosses between single mutants to generate complete null genotypes.
**Table S1** List of primers used for tetra primers PCR.
**Table S2** List of primers used in qRT‐PCR.
**Table S3** RVA and hardness parameters.
