# Supplementary material for: Combining mutations at genes encoding key enzymes involved in starch synthesis affects the amylose content, carbohydrate allocation and hardness in the wheat grain
Source: Plant Biotechnol J. 2018 Apr 17;16(10):1723–34. doi: 10.1111/pbi.12908 (PMC6131419; doi:10.1111/pbi.12908)
Supplement: Supplementary file 1 — Figure S1 Scheme of crosses between single mutants to generate complete null genotypes. Table S1 List of primers used for tetra primers PCR. Table S2 List of primers used in qRT‐PCR. Table S3 RVA and hardness parameters. [file PBI-16-1723-s001.pdf]

Table S1 List of primers used in TETRA- ARMS PCR

Mutant	Inners 5'-3'	Outers (5'-3')	T. annealing (°C)	Amplicon size (bp)		
				Common	W.T.	Mutant
**SBEIIa-A^{-1}**	F-GCAAATGCAGGAGAAGTATGTCTGCCC<br>R-TCTGGTCGTTTAGGTTGAGGATGGTA	F-CAACCATGGTGATGTTTGTATGCTTG<br>R-GCCTGCCTTCTGCCCTGTG	64	587	457	175
**SBEIIa-B^{-1}**	F -TGCAGCATTAGTAGGTGACTTCAACAAGTA<br>R- GGTCATAGTATCTGCATTTGGATGCC	F-CTGGAGCGCATGTACGTCTTAAC<br>R-CACCATAATCATCCTGAAAAGATCG	61	581	206	430
**SBEIIa-D^{-1}**	F-GAATGCTCCTGGATTGCCATTATATG<br>R-TAAAAGGCTTGGATACAATGCAGGGT	F- GAGGCAGTGGGCATGTGAAAGTCAT<br>R-GTGCATGAAGCATTAGGATGTAATAG	64	617	522	146

The table reports the combination of the four primers (two inners and two outers) used to follow the mutations SBEIIa-A^{-1}, SBEIIa-B^{-1} and SBEIIa-D^{-1} identified in Botticella et al 2011. On the right side of the table it is reported the pattern of amplicons analyzed by electrophoresis on agarose gel. The amplicon designed as " common" is obtained both for mutant and wild type genotype.

Table S2 List of primers used in qRT-PCR

Category	Gene	oligo (5'-3')	Reference
Sugar mobilization	***Susy2***	F GTGTGTCCGGCTACCACAT<br>R AGCTTCCAGGTGTACTTCTCCTC	this work
	UGPase	F TTGCGAACTTCAATGACAGAC<br>R GTGCCCTACGAGACCCT	Yu et al. 2014
	ADPG-t	F ggccatcgagcacttcacttac<br>R ataggtgcacagggttgagg	this work
Starch	***AGP-L1***	F GCCCCTGTTGGAGAGAGCCG<br>R TAGCAGGGTCGTCGATGGCG	Kang et al. 2014
	AGP-L2	F ATTGATGGCAGCCGGGCGTC<br>R TCGGTAAGCCGAGGAGAGTGGT	
	AGP-S1-a	F CCTTCCAAGCGTGAACAA<br>R TTCCGAGAACACTATCATCAAC	
	AGP-S2	F TAATTCCGAGCGGGACAG<br>R AAATGGTGCCTTGAGTGG	
	GBSSI	F CGGCATGGACGTCAGCGAGT<br>R AGGGGCACCTTCCGGTCCAC	
	SSI	F GAACTGGGGGCCTCCGAGACA<br>R CCTCCCAGGACGGCTTGTGC	
	SSII	F CACGCCGGTGAACGGTGAGA<br>R CGGCGAGGCGACGTTAGCTT	
	SSIII	F CGGTTGACGAAGTCGGGCCT<br>R CCCAGCTTCATCCACTGCACCC	
	SSIV	F CGACGAGCAGGGCCTAAGCA<br>R AGCCGAGGTGTCCCAGCTGAA	
	SBEI	F TGGGTCGATCGGGTTCCTGCAT	

		R ACGTGGAGCGTCAGGCTTTCG	
	SBEIIa	F GCAAGTCCGGCGCAACCTGA<br>R CTCGCGGTTTCTCCCCCACG	
	SBEIIb	F CGCCTTCCATCGACGGTCCC<br>R TCCCGGTGGTGGCAGAATGC	
	ISAI	F AGTACCGGGACATTGTGCGCC<br>R GCCAAGGTTTCCTTCCTCCTGCC	
	ISAII	F GCCACGTCTGGCACGTCTCG<br>R TGGCAGCAACCAGGTCACCG	
	PulI	F CCCTCCTCAGTCCCAGGTGT<br>R TGCGGACACACATTGAAGACCG	
Arabinoxylans	***GT-43***	F TGGTGTCGGAGGATTATAGCA<br>R GTATAGGCAGCAGCTTGTTCG	Marcotuli et al. 2016
	GT-47	F GGACATACTGAGGAAGCAAAGG<br>R AGATGCTACCACGGCTTCAG	
	GT-61	F GCTGAGCGAGCAGTACCC<br>R GCTCAGTTGTTGGCCTTGG	
	GT-61-2	F AGCCTCACCGACCAGTACC<br>R CAATGGCGGATCAGTAGTTCT	
β-glucans	***CSLF6***	F GCAAGACGCCAGTCGTGG<br>R CAGTTGTAGTACCCTGCGTCGA	Nemeth et al. 2010
Ta2526	***house-keeping***	F CGAGATCGACCAAGAATGG<br>R TGAGTGTTGCCTCCCTCC	Nemeth et al. 2010

Table S3. RVA and Hardness parameters

RVA					Hardness				
**Genotype**	**PV**	**FV**	**BD**	**SB**	**Seeds n°**	**Distribution %**			
						**≤ 33**	**> 33 ≤ 46**	**> 46 ≤ 59**	**> 59**
Cadenza	248.1±3.1	121.7±0.7	277.4±4.4	152.1±1.0	300	5	13	28	54
Cad-GBSSI*	282±3.7	101.75±3.8	180±4.3	160.5±1.1	300	0	2	11	87
Cad-SSIIa*	10.7±3.6	9.8±3.2	34.3±4.5	25.2±0.2	300	0	0	6	94
Cad-SBEIIa*	22.8±3.0	19.6±2.5	51.7±4.5	31.7±1.1	300	0	1	6	93

PV: Peak viscosity, FV: Final Viscosity; BD: Break Down, SB: Setback.

Seeds n°: number of seeds analyzed by SKCS;Distribution % indicates the distribution % of hardness index

a

b

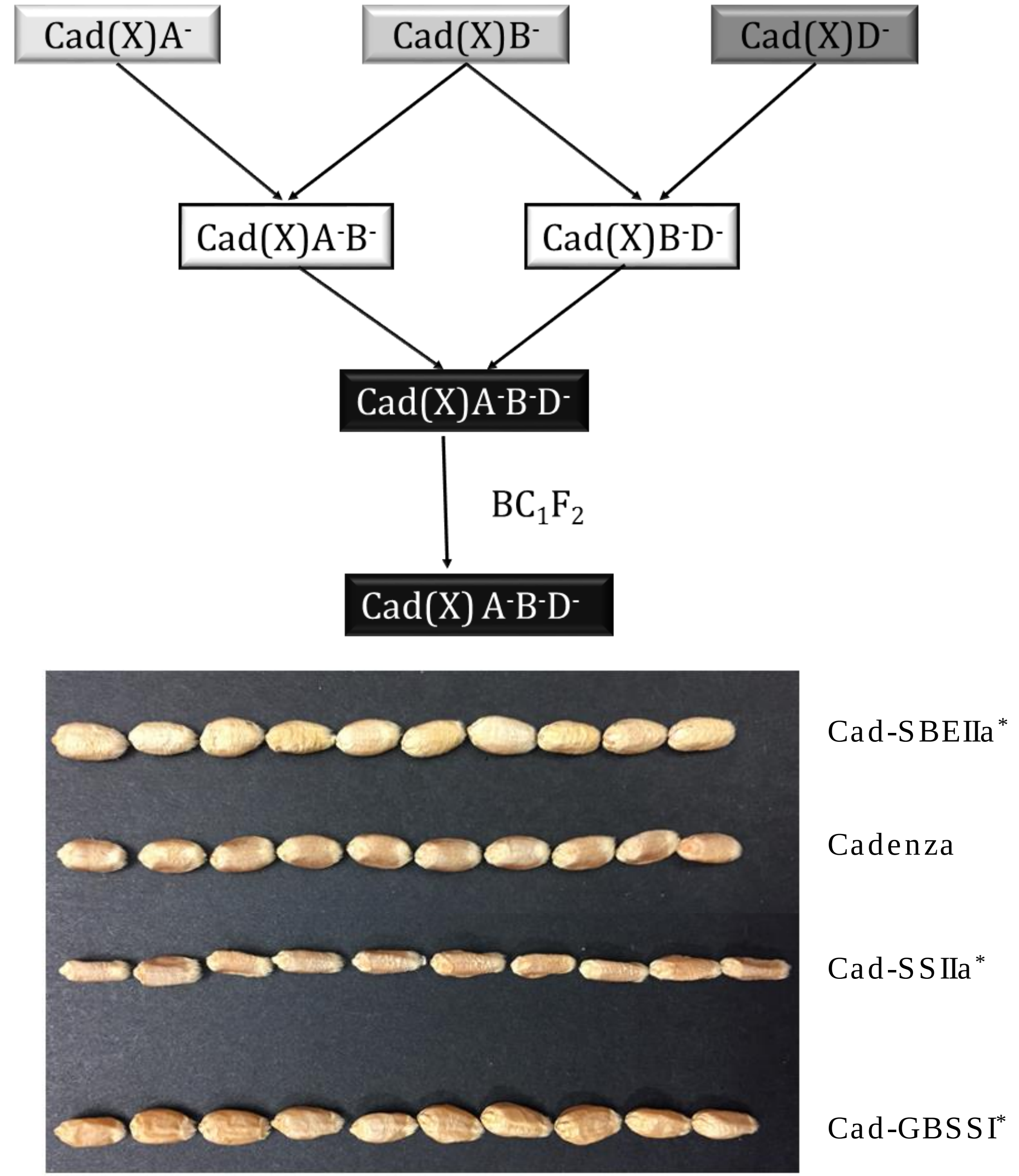

Fig. S1 (a) Scheme of crosses executed to pyramid null mutations of the three homoeologus for each gene (X, alternatively standing for SBEIIa, GBSSI and SSIIa; Cad -Cadenza). (b) Picture showing seed morphology of the mutant lines along with the control.
